# Analysis of lncRNA-miRNA-mRNA Interactions in Hyper-proliferative Human Pulmonary Arterial Smooth Muscle Cells

**DOI:** 10.1038/s41598-019-46981-4

**Published:** 2019-07-19

**Authors:** Mahendran Chinnappan, Sumedha Gunewardena, Prabhakar Chalise, Navneet K. Dhillon

**Affiliations:** 1 Division of Pulmonary and Critical Care Medicine, University of Kansas Medical Center, Kansas City, Kansas USA; 2 Department of Molecular & Integrative Physiology, University of Kansas Medical Center, Kansas City, Kansas USA; 3 Kansas Intellectual and Developmental Disabilities Research Center, University of Kansas Medical Center, Kansas City, Kansas USA; 4 Department of Biostatistics, University of Kansas Medical Center, Kansas City, Kansas USA

**Keywords:** Non-coding RNAs, Molecular medicine

## Abstract

We previously reported enhanced proliferation of smooth muscle cells on the combined exposure of HIV proteins and cocaine leading to the development of HIV-pulmonary arterial hypertension. Here, we attempt to comprehensively understand the interactions between long noncoding RNAs (lncRNAs), mRNAs and micro-RNAs (miRNAs) to determine their role in smooth muscle hyperplasia. Differential expression of lncRNAs, mRNAs and miRNAs were obtained by microarray and small-RNA sequencing from HPASMCs treated with and without cocaine and/or HIV-Tat. LncRNA to mRNA associations were conjectured by analyzing their genomic proximity and by interrogating their association to vascular diseases and cancer co-expression patterns reported in the relevant databases. Neuro-active ligand receptor signaling, Ras signaling and PI3-Akt pathway were among the top pathways enriched in either differentially expressed mRNAs or mRNAs associated to lncRNAs. HPASMC with combined exposure to cocaine and Tat (C + T) vs control identified the following top lncRNA-mRNA pairs, ENST00000495536-HOXB13, T216482-CBL, ENST00000602736-GDF7, and, TCONS_00020413-RND1. Many of the down-regulated miRNAs in the HPASMCs treated with C + T were found to be anti-proliferative and targets of up-regulated lncRNAs targeting up-regulated mRNAs, including down-regulation of miR-185, -491 and up-regulation of corresponding ENST00000585387. Specific knock down of the selected lncRNAs highlighted the importance of non-coding RNAs in smooth muscle hyperplasia.

## Introduction

Human immunodeficiency virus (HIV)-1 associated pulmonary arterial hypertension (PAH) is one of the prominent noninfectious complications of HIV infection^1,2^. Since, not all the HIV infected patients develop HIV-PAH, it is proposed that other environmental factors may serve as second hit in the development of PAH in these individuals^3,4^. Reports suggest that drugs of abuse such as cocaine, opioids/morphine and methamphetamine are possible independent risk factors for the development of pulmonary arterial hypertension (PAH)^5–8^. Adding to this, the intravenous drug usage (IVDU) is widely reported to be a major risk factor for HIV infection and reports including from our lab suggest that IVDU and HIV-1 infection act in concert in potentiating PAH^9–15^.

PAH is a pathological condition caused by pulmonary vascular remodeling involving hyper-proliferation of pulmonary arterial smooth muscle cells (PASMC) and endothelial cell (EC) dysfunction leading to increased blood pressure in the arteries leading to lung^16,17^. Both SMC and EC are not infected by HIV-1 but are continuously exposed to the viral proteins that are secreted by the infected immune cells of the host. Various studies, including from our group, have reported the role of viral proteins such as negative regulatory factor (Nef), glycoprotein-120 and trans-activator of transcription (Tat) in endothelial injury and pulmonary vascular remodeling^7,18–20^. Furthermore, our previous findings based on cell-culture and non-infectious HIV-transgenic model expressing 7 of 9 viral proteins demonstrate hyper-proliferation of pulmonary SMC in response to HIV protein(s) and cocaine^21^. We also observed that while HIV proteins namely Tat, gp120 and Nef were equally able to down-regulate the expression of bone morphogenetic protein receptors (BMPR), HIV-Tat demonstrated maximum effect in the presence of cocaine in human PASMCs^22^.

Multiple molecular mechanisms including pathways regulated by noncoding RNAs have been reported to contribute to the development of PAH^23,24^. The significance of noncoding RNAs can be realized by the facts that only 2% of the human genome codes for protein coding genes and 90% of the rest of 98% of the genome codes for these non-protein coding genes^25^. Non-coding RNAs such as micro RNAs (miRNAs) and long non-coding RNAs (lncRNAs) play critical roles in regulating the fundamental aspects of cellular homeostasis and tissue development^26,27^. miRNAs are generally short RNAs with 21–23 nucleotides (nts) that regulate gene expression post transcriptionally by binding to mRNAs in a sequence specific manner. We had earlier characterized a novel role for miR-216 and -301 in modulating HIV-protein Tat and cocaine mediated SMC hyperplasia via targeting BMPR-2 mRNA^10^. Our studies also revealed the role of miRNAs carried in extracellular vesicles derived from HIV-infected and morphine exposed macrophages in regulating proliferation of PASMCs^11^.

LncRNAs, novel class of non-coding RNAs that were recently discovered with the advancement of RNA-sequencing techniques are longer than 200 nts^28^. While the roles of miRNAs in PAH have been elaborately studied, functions of lncRNAs in smooth muscle dysfunction and PAH remain less explored. lncRNAs are reported to regulate gene expression by multiple mechanisms. Some function as decoy, guide or scaffold to a diverse set of transcription factors and chromatin silencing proteins and therefore regulate expression of protein coding genes at the transcriptional and post-transcriptional levels^28^. The lncRNAs sharing common miRNA bindings sites as target mRNA can also act as competitive endogenous RNA (ceRNA) by functioning as miRNA sponge or they can also act as precursors and encode miRNAs therefore regulating the levels and downstream functions of miRNAs^29^. Since multiple ways of interaction between miRNAs to lncRNAs, miRNA to mRNA, lncRNA to mRNA (cis and trans) have been reported to play crucial roles in determining the cellular fate, it becomes essential to study these interactions in an integrated fashion to comprehensively decipher the factors underpinning the pathology of PAH.

Here, we report the first integrated analysis of altered lncRNA, miRNA and mRNA expression in hyper-proliferative human primary PASMCs treated with cocaine and Tat uncovering the critical interactions between these molecules that may eventually help us not only in understanding the cocaine and Tat induced hyper-proliferation but also in general regulation of smooth muscle dysfunction associated with all types of pulmonary hypertension.

## Results

### Differential expression of lncRNAs in Cocaine and Tat protein treated HPASMCs

**Figure 1:**
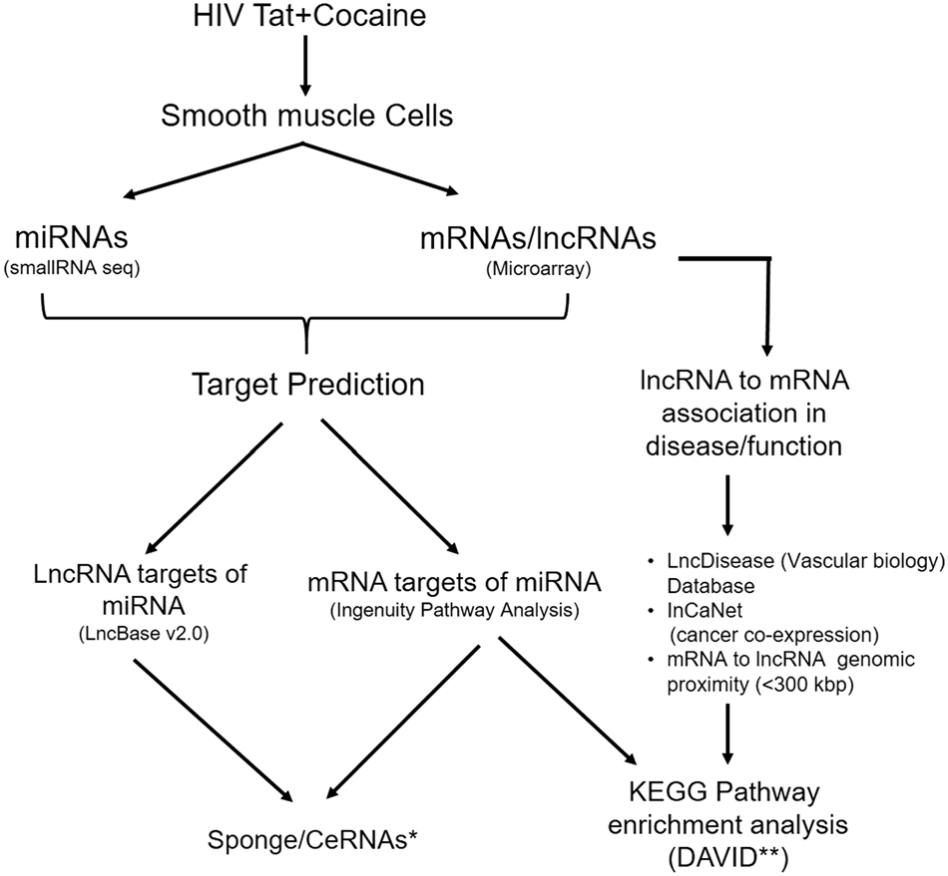
Schematic showing the overall workflow of the analysis. *Competing endogenous RNA; **The Database for Annotation, Visualization and Integrated Discovery (DAVID) v6.8.

**Figure 2:**
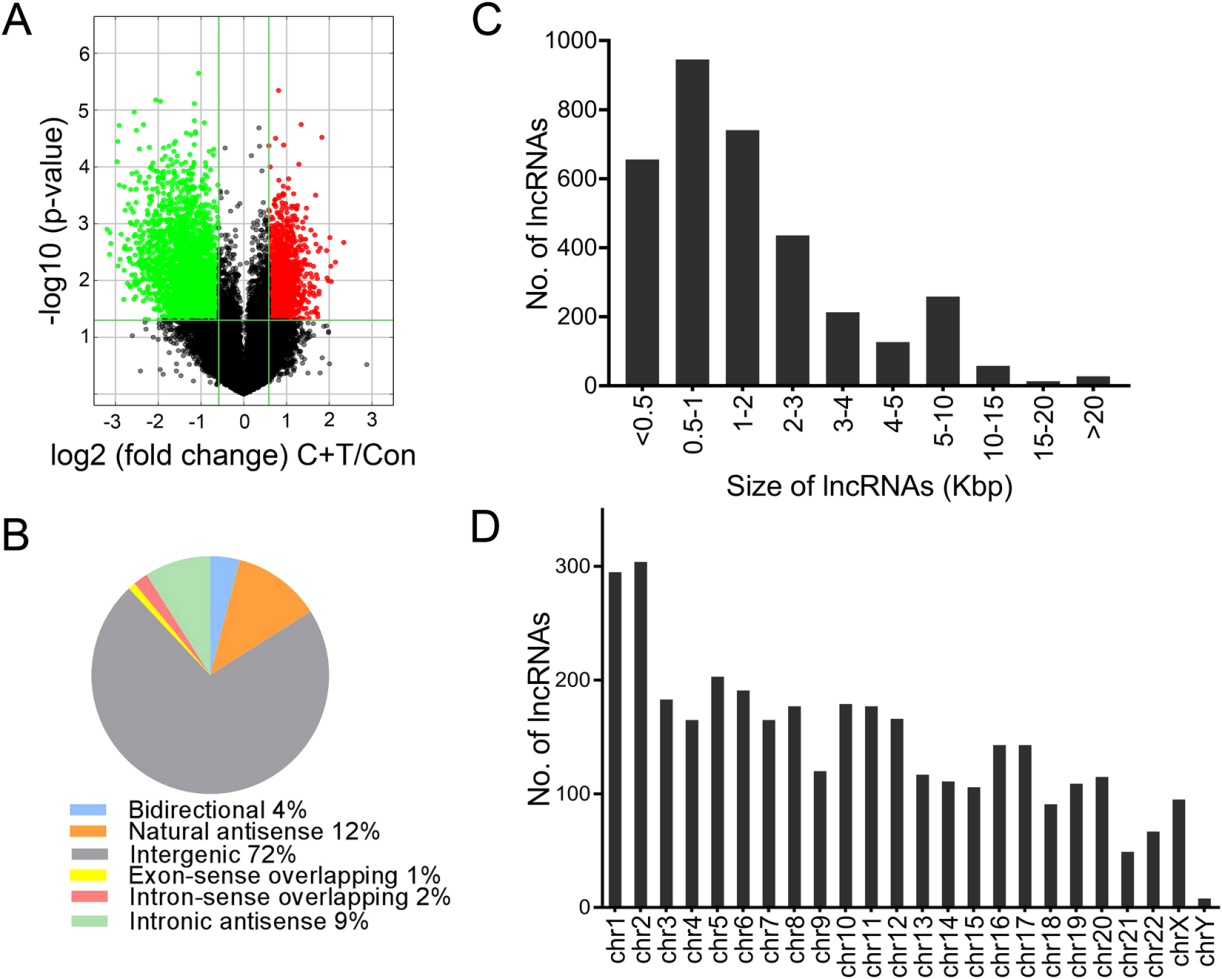
Differential expression and characteristics of lncRNAs. (**A**) Volcano plot for significantly dys-regulated lncRNAs in Cocaine and Tat (C + T) treated HPASMCs when compared with untreated control (Con). Subclasses (**B**), size ranges (**C**) and chromosome location (**D**) of significantly dys-regulated lncRNAs in C + T vs control group.

As shown in the schematic diagram in Fig. 1, microarrays were used to capture the expression profile of lncRNAs in Tat and cocaine treated HPASMCs. This analysis revealed a number of lncRNAs with significant differential expression (Fig. 2A). A total of 1,291 lncRNAs were significantly up-regulated and a total 2,155 were down-regulated in the combined treatment of cocaine and Tat (C + T) compared to control. LncRNAs have been classified into many different groups based on their genomic location with relevance to the nearby protein coding gene sequences^30^ which helps to predict some of their possible mode of action in regulating other cellular RNA molecules. Based on this classification, significantly differentially expressed lncRNAs in the C + T group compared to the untreated control were found to be 72% (2,509) intergenic, 12% (415) natural antisense and 9% (321) intronic antisense regions (Fig. 2B) in the genome. These lncRNAs ranged from 100 to 43,681 nts in size (Fig. 2C) and were distributed across all chromosomes (Fig. 2D).

### Differential expression of mRNAs in Cocaine and Tat protein treated HPASMCs

**Figure 3:**
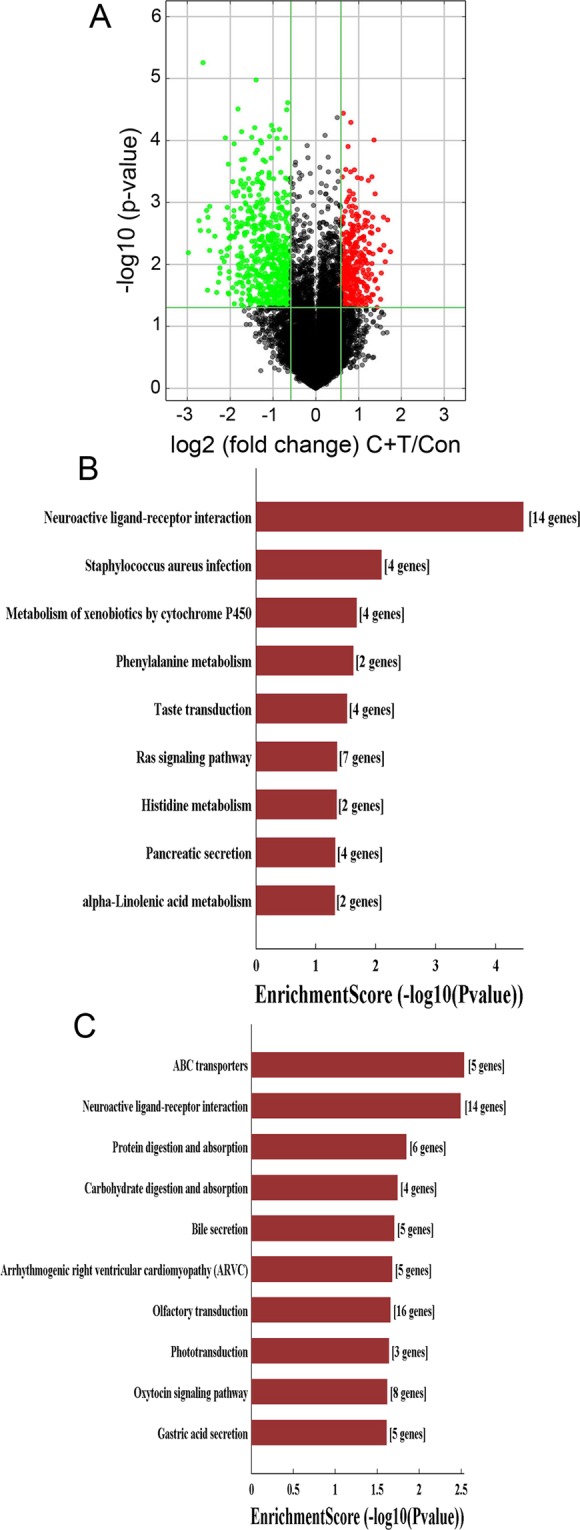
Differential expression and pathway analysis of dys-regulated mRNAs in cocaine and Tat (C + T) treated HPASMCs. (**A**) Volcano plot for significantly dys-regulated mRNAs in C + T treated group vs. control. KEGG pathway enrichment for up-regulated (**B**) and down-regulated (**C**) mRNAs in cocaine and Tat (C + T) treated HPASMCs.

Analysis of microarray data of mRNA expression levels in the combined treatment of cocaine and Tat resulted in the up-regulation of 325 mRNAs and down regulation of 536 mRNAs, compared to control (Fig. 3A). A biological functional and pathway analysis based on GO terms and KEGG pathways was performed in order to understand the relevance of the significantly differentially expressed mRNAs to the phenotypic changes in HPASMC upon combined treatment of cocaine and Tat (Fig. 3 and Tables 1 and 2). For both, up-regulated (Fig. 3B) and down regulated (Fig. 3C) mRNA, the neuroactive ligand receptor pathway appeared as one of the top most enriched KEGG pathways. The up-regulated genes in this pathway were, *DRD2*, *GLP1R*, *HTR1A*, *OPRL1* and *PTGER3*, and, the down-regulated genes were, *P2RY13*, *PRLR*. These genes are involved in G-protein coupled receptor signaling, and GLP1R is being recommended for PAH therapeutics^31^. The Ras signaling pathway, which was significantly enriched among the up-regulated genes in cocaine + Tat treated cells, had seven genes: *EFNA3*, *FGF22*, *FGF3*, *FGFR2*, *PLA2G2A*, *PLA2G5*, and, *RASGRP4*, associated with it (Fig. 3B), and of these the FGF signaling molecules are well known to induce proliferation^32^. The most highly enriched biological process associated GO term of the differentially expressed genes was calcium homeostasis for up-regulated genes (Table 1) and G-protein coupled receptor signaling for down-regulated genes (Table 2). The most enriched molecular function associated GO term for both the up and down regulated genes was trans-membrane receptor activity. The GO term of the cellular component most significantly associated with both the up and down regulated genes was intrinsic component of membrane.

**Table 1:** GO terms for up-regulated mRNAs in HPASMCs treated with cocaine and Tat compared to untreated control.

GO.ID	Term	Count
***Biological Process***		
GO:0055074	calcium ion homeostasis	18
GO:0072507	divalent inorganic cation homeostasis	18
GO:0051480	regulation of cytosolic calcium ion concentration	14
GO:0003008	system process	50
GO:0050877	neurological system process	36
GO:0006874	cellular calcium ion homeostasis	16
GO:0030802	regulation of cyclic nucleotide biosynthetic process	9
GO:0055065	metal ion homeostasis	20
GO:1900371	regulation of purine nucleotide biosynthetic process	9
GO:0030808	regulation of nucleotide biosynthetic process	9
***Cellular Component***		
GO:0031224	intrinsic component of membrane	113
GO:0016021	integral component of membrane	109
GO:0005887	integral component of plasma membrane	43
GO:0031226	intrinsic component of plasma membrane	43
GO:0044425	membrane part	123
GO:0005886	plasma membrane	98
GO:0071944	cell periphery	99
GO:0044459	plasma membrane part	55
GO:0098590	plasma membrane region	22
GO:0001673	male germ cell nucleus	2
***Molecular Function***		
GO:0099600	transmembrane receptor activity	38
GO:0004872	receptor activity	44
GO:0060089	molecular transducer activity	44
GO:0004888	transmembrane signaling receptor activity	36
GO:0005509	calcium ion binding	23
GO:0038023	signaling receptor activity	37
GO:0004930	G-protein coupled receptor activity	25
GO:0022836	gated channel activity	13
GO:0005231	excitatory extracellular ligand-gated ion channel activity	5
GO:0010181	FMN binding	3

**Table 2:** GO terms for down-regulated mRNAs in HPASMCs treated with cocaine and Tat compared to untreated control.

GO.ID	Term	Count
***Biological Process***		
GO:0007186	G-protein coupled receptor signaling pathway	50
GO:0055085	transmembrane transport	50
GO:0034220	ion transmembrane transport	39
GO:0045665	negative regulation of neuron differentiation	13
GO:0007283	spermatogenesis	24
GO:0048232	male gamete generation	24
GO:0006811	ion transport	52
GO:0031345	negative regulation of cell projection organization	11
GO:0010977	negative regulation of neuron projection development	10
GO:0051961	negative regulation of nervous system development	15
***Cellular Component***		
GO:0031224	intrinsic component of membrane	176
GO:0031226	intrinsic component of plasma membrane	69
GO:0044425	membrane part	197
GO:0016021	integral component of membrane	164
GO:0071944	cell periphery	158
GO:0005886	plasma membrane	155
GO:0044459	plasma membrane part	91
GO:0005887	integral component of plasma membrane	61
GO:0005891	voltage-gated calcium channel complex	5
GO:0031225	anchored component of membrane	10
***Molecular Function***		
GO:0099600	transmembrane receptor activity	58
GO:0004872	receptor activity	65
GO:0060089	molecular transducer activity	65
GO:0004888	transmembrane signaling receptor activity	54
GO:0038023	signaling receptor activity	56
GO:0004930	G-protein coupled receptor activity	38
GO:0043225	ATPase-coupled anion transmembrane transporter activity	4
GO:0004871	signal transducer activity	61
GO:0030246	carbohydrate binding	16
GO:0008514	organic anion transmembrane transporter activity	8

### In-silico functional association between differentially expressed lncRNAs and mRNAs in cocaine and Tat treated HPASMCs

**Figure 4:**
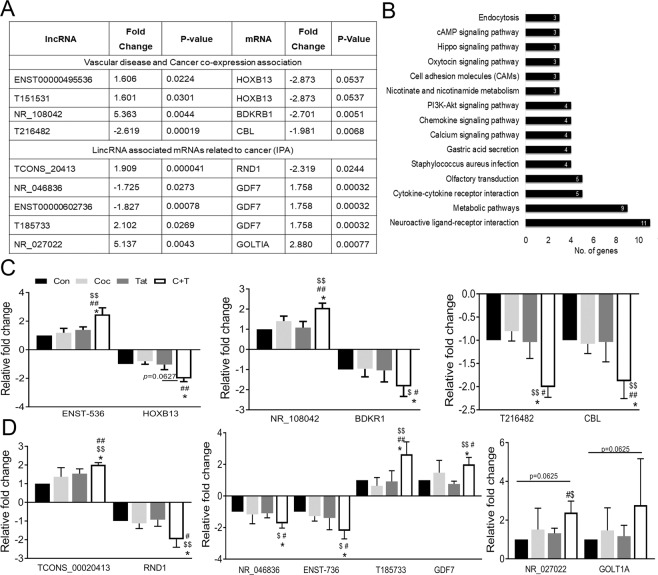
Predicted association of dys-regulated LncRNAs to altered mRNA expression in cocaine and Tat treated HPASMCs. (**A**) Table lists microarray details of selected top lncRNA/mRNA predicted associations. (**B**) KEGG pathway enrichment for mRNAs associated to lncRNAs based on genomic proximity (<300kbp). (**C**,**D**) Quantitative RT-PCR based validation of selected lncRNA/mRNA pairs from vascular disease and cancer co-expression database (**C**); and from genomic proximity (**D**) analysis. *p*-value ^*^<0.05 vs. control (Con), ^##^<0.01, ^#^<0.05 vs. cocaine(Coc), ^$$^<0.01, ^$^<0.05 vs. Tat.

We looked for associations between the significantly differentially expressed mRNAs and lncRNAs in the relevant databases mentioned in Methods, to find out the reported association between them along with their known functional significance. We investigated the association of all significantly altered lncRNA and mRNAs in the C + T group compared to control using the LncDisease database and found that 37 significantly dys-regulated lncRNAs, were related to multiple differentially expressed mRNAs associated with cardiovascular diseases. The up-regulated lncRNA ENST00000495536 was one of the top candidates in the list and was associated with down-regulated HOXB13 mRNA in cocaine + Tat group (Fig. 4A).

We next performed a correlation analysis of the significantly differentially expressed lncRNA and mRNA in the C + T group in conjunction with the lncRNA Cancer database. Significantly differentially expressed lncRNAs were associated with significantly differentially expressed mRNAs that showed a differential expression pattern that was in concordance with the correlation information obtained from the lncRNA Cancer database. We identified associations between 19 unique lncRNAs and 16 unique cancer related genes, making a total of 27 lncRNA to mRNA pairs that matched co-expression correlation values in the range of 0.6 to 0.8 in the lncRNACancer database (Supplementary File I). Cancer driver genes namely CBL, GFI1, RASGRF2 and ELK3 were significantly down-regulated and associated with the significantly down-regulated lncRNAs -T216482, -T334719, -T217484 and -T265137 respectively. On the other hand, down-regulated HOXB13 in C + T group was associated with the up-regulated lncRNA T151531. Figure 4A, lists some of the top lncRNA and mRNA associations retrieved from this analysis and suggests underlining interactions between these RNAs in regulating critical proliferation signals in HPASMC.

Given that lncRNAs are known to regulate the expression of proximal protein coding genes in *cis* regulatory mechanisms^33^, we explored the lncRNA and mRNA microarray data to identify lncRNAs that may regulate in cis nearby mRNAs. A functional analysis of these mRNAs using the DAVID annotation tool revealed their association to multiple proliferation related pathways such as Hippo, PI3-Akt and cAMP signaling (Fig. 4B). A list of, long intergenic noncoding RNAs (lincRNAs) or antisense lncRNAs, along with their associated mRNA was prepared for the significantly differentially expressed lincRNAs and antisense lncRNAs in the C + T group based on their genomic proximity (<300 kbp from each other). A total of 345 pairs of lincRNA - mRNA associations were found. The lncRNA TCONS_00020413 was one of the top candidates in this list and was up-regulated in C + T compared to control, while expression of its nearby gene, RND1, was significantly down regulated (Fig. 4A). RND1 is a tumor suppressor protein that belongs to the Rho family GTPases. A biological functional analysis of these significantly differentially expressed mRNAs, in the lincRNA/mRNA pairs, done using ingenuity pathway analysis (IPA), listed cancer as one of the top diseases, with 208 mRNAs out of 345 being associated with it. GDF7 was one of the top candidate genes among the cancer related molecules. It was associated with three lncRNAs namely, T185733, NR_046836 and ENST00000602736 (Fig. 4A). The analysis of significantly differentially expressed antisense lncRNA and significantly differentially expressed mRNA, that were in close proximity to each other, yielded 21 lncRNA/mRNA pairs. The up-regulated antisense lncRNA T041024 in C + T group was in proximity to the down-regulated ALOX5 gene implicated in hypertension.

In order to validate the microarray expression data of the identified lncRNA/mRNA pairs, we performed qRT-PCR on the top ranked lncRNAs that had a raw intensity above 100 (raw intensity is a surrogate measure of gene expression in microarrays) and their associated mRNAs from each of the three data sets mentioned above (Fig. 4A). From the lncRNA/mRNA associations established using the vascular disease database, we confirmed the up-regulation of the lncRNA, ENST00000495536, which was 2.4-fold up-regulated in cocaine + Tat treated HPASMCs compared to untreated cells. This lncRNA was associated with the HOXB13 gene, which was 2.1-fold down-regulated in cocaine + Tat. These alterations in the cocaine + Tat treated cells were also significant when compared to HPASMCs treated with only cocaine or Tat mono-treatments. Furthermore, mono- treatment of cocaine or Tat resulted in no significant change in either ENST00000495536 or HOXB13 mRNA levels compared to un-treated controls (Fig. 4C). We also observed that the lncRNA and mRNA pair NR_108042-BDKR1 was up or down-regulated ~2 -fold, respectively, in the cocaine + Tat group compared to control. From the lncRNA and mRNA associations obtained by cancer co expression analysis, we validated by qRT-PCR, the differential expression of lncRNA T216482 (2 fold down-regulated) and its associated mRNA, CBL (1.9 fold down-regulated) in C + T treated cells compared to control (Fig. 4C). These changes were also similarly significant when C + T treated cells were compared to cells with the mono-treatment of cocaine or Tat.

We also validated by qRT-PCR, the expression of lincRNA TCONS_00020413 identified from the genome proximity analysis (Fig. 4A) and its associated gene, RND1. This lncRNA was significantly up-regulated by 2-fold in C + T treated HPASMC compared to control, whereas mono-treated cells with either cocaine or Tat resulted in only a non-significant 1.3 fold and 1.5 fold up-regulation, respectively (Fig. 4D). Its associated mRNA, RND1, was 2-fold down-regulated in cells treated with the cocaine + Tat (Fig. 4D). Further, we validated the mRNA expression of GDF7 (BMP12) and the three lincRNAs associated with it that was identified by their close genomic proximity. Validation by qRT-PCR established a 2-fold up-regulation of GDF7 while its associated lncRNA T185733 was 2.8-fold up-regulated and the other two lncRNAs, NR_046836 and ENST00000602736, were 1.7-fold and 1.8-fold down-regulated respectively, in cocaine + Tat compared to control (Fig. 4D). One of the lncRNA - mRNA pairs that was identified from the microarray analysis and selected for validation by qRT-PCR, NR_027022 – GOLTIA, showed no significant difference in expression in cocaine + Tat treated cells when compared to  untreated control (Fig. 4D).

### Dys-regulation of miRNAs in cocaine and Tat protein treated HPASMCs

**Figure 5:**
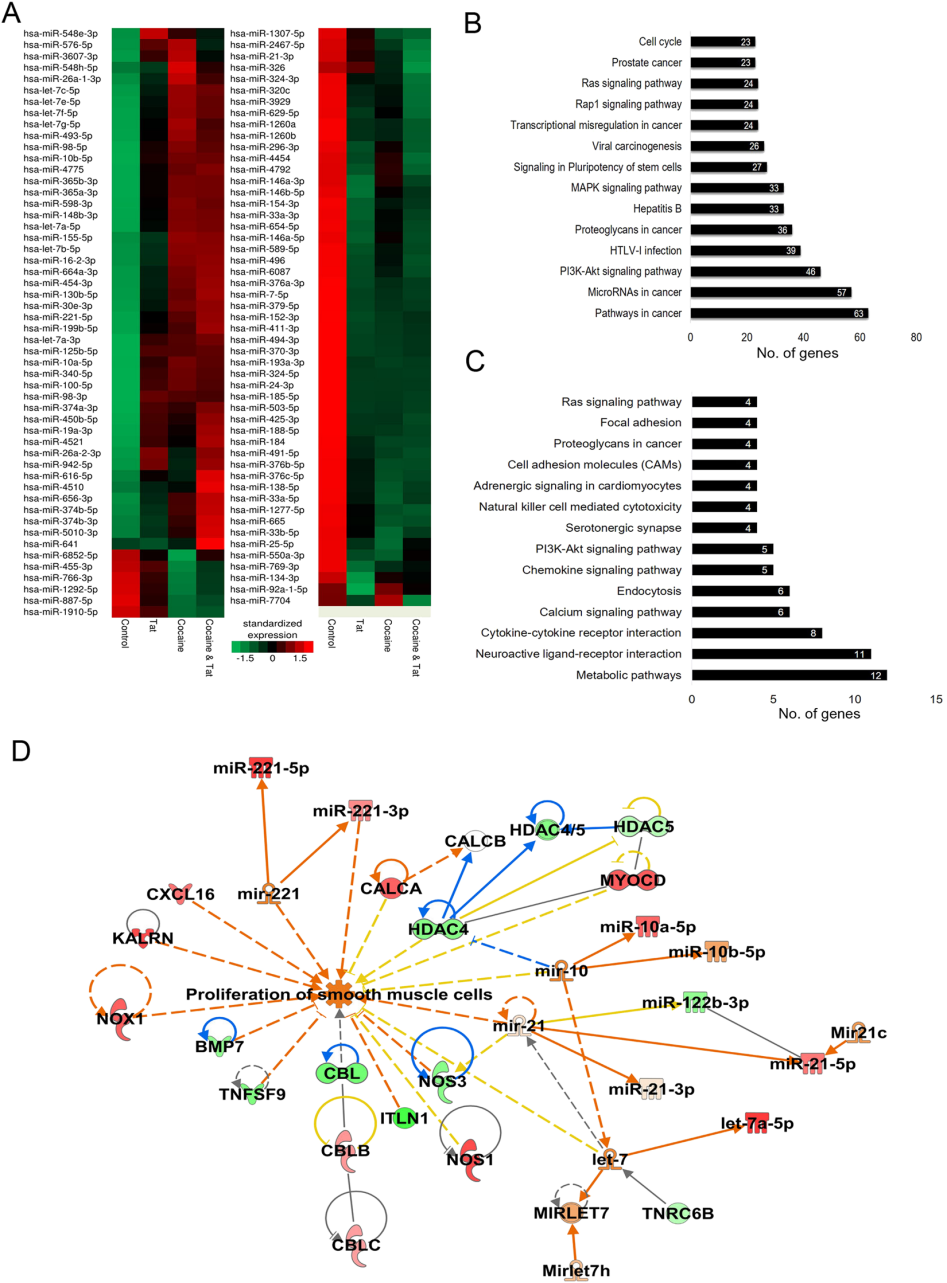
Differential expression of miRNAs and their predicted and experimentally validated mRNA targets in HPASMCs treated with cocaine and/or Tat. (**A**) Hierarchical clustering for differentially expressed miRNAs. (**B**,**C**) Biological functional analysis of significantly dysregulated miRNAs using DAVID tool on all the known down-stream mRNA targets of these miRNAs based on IPA analysis (**B**) and including only significantly differentially expressed mRNA target genes in the microarray data (**C**). (**D**) Combined functional analysis of the significantly differentially expressed mRNA and miRNAs in association with SMC proliferation using IPA (QIAGEN Inc., https://www.qiagenbioinformatics.com/products/ingenuitypathway-analysis).

**Figure 6:**
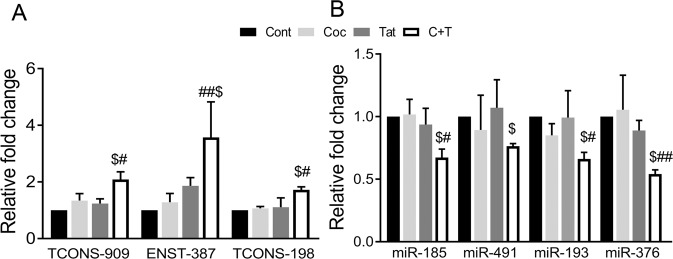
Quantitative RT-PCR analysis of the selected up-regulated lncRNA (**A**) and down-regulated miRNA (**B**) in the cells treated with and without cocaine and/or Tat. *p*-value ^##^<0.01, ^#^<0.05 vs. cocaine (Coc); ^$^<0.05 vs. Tat.

To determine the miRNAs that are involved in Tat and cocaine mediated hyper-proliferation of smooth muscle cells, we performed small RNA sequencing on total RNA isolated from HPASMC treated with cocaine and Tat for 48 h. Analysis of significantly differentially expressed miRNAs revealed 78 miRNAs in the cocaine + Tat group, 48 in the Tat only group and 60 in the cocaine only group, when compared with untreated control cells (Fig. 5A). In order to determine the genes that are regulated by these miRNAs and ascertain their functional relevance, we used the IPA miRNA target filtering tool to identify all the known down-stream target genes of these miRNAs and performed a biological functional analysis on them using DAVID (Fig. 6B). We next filtered these genes to only include those genes that were significantly differentially expressed in our microarray data and performed a biological functional analysis on them (Fig. 5C). The above analysis was performed for miRNAs and mRNAs that were significantly differentially expressed in cocaine + Tat group compared to control. The functional analysis results from DAVID on all down-stream mRNA targets of the significantly differentially expressed miRNAs suggest that these miRNAs may regulate mRNAs related to cancer and proliferation related pathways such as PI3-Akt, Ras, MAPK (Fig. 5B). Furthermore, the functional analysis results from DAVID on the significantly differentially expressed down-stream mRNA targets of the significantly differentially expressed miRNAs revealed associations to various proliferation related pathways such as PI3-Akt, Ras and calcium signaling (Fig. 5C).

In addition, a combined functional analysis of the significantly differentially expressed mRNA and miRNAs in cocaine + Tat treated HPASMCs using IPA highlighted several PAH related factors that support smooth muscle proliferation (Fig. 5D). Among the identified targets were the down-regulated genes, BMP7, CBL, TNFSF9, ITLN1, Nos3, TNRC6B, HDAC, and the up-regulated genes, CALCA, NOX1, KALRN, CXCL16, MYOCD and NOS1, which are known to induce smooth muscle cell proliferation. The analysis also revealed the significantly differentially expressed miRNAs in our data, miR-221, -10a, -122b, -21 and let-7, which are reported to activate proliferative signals in smooth muscle cells (Fig. 5D). Overall, the dysregulated miRNA and mRNA profile in cocaine + Tat smooth muscle cells shows that they may play a critical role in cocaine + Tat induced hyper- proliferation of HPASMC.

### Integrated analysis of significantly dys-regulated lncRNA, miRNA and mRNA in HPASMC exposed to combined treatment of cocaine and Tat

Long noncoding RNAs are known to function as competing endogenous RNAs (ceRNAs) and affect mRNA levels by sequestering common miRNAs that target both the lncRNA and mRNAs^34^. We explored this function of ceRNA by analyzing the association of the up-regulated lncRNAs and mRNAs in our microarray data to the down-regulated miRNAs from our small RNAseq data for the HPASMCs exposed to the combined treatment of cocaine and Tat. Since lncRNAs with high expression levels are reported to function as sponge RNAs much effectively than lncRNAs with comparatively low expression^35^, we selected those lncRNAs that were significantly up-regulated with raw signal intensity greater than 100, for this analysis. For reasons of annotation, only those lncRNAs that were listed in any one or more of the public databases, GENCODE and Ensemble or described by Cabili *et al*.^36^, were selected. We used LncBase v.2 available in DIANA Tools to retrieve information on miRNA binding to specific lncRNAs. The LncBase v.2 database holds information for over 70 k miRNA to lncRNA interactions, supported by either direct or indirect experiments derived from manually curated publications, and the analysis of 153 Argonaut-crosslinking immunoprecipitation (AGO CLIP)-Seq libraries^37^. We used IPA to ascertain the experimentally validated miRNA to mRNA interactions.

Out of the selected up-regulated lncRNAs, 10 of them had one or more binding sites for 12 of the down-regulated miRNAs. These 12 miRNAs targeted 134 of the up-regulated mRNAs. Using this information, we generated a putative molecular interactive network describing how lncRNAs acting as ceRNAs could potentially regulate mRNA expression by modulating the level of its up-stream miRNA regulator(s) (Table 3). This analysis indicates that some of the up-regulated lncRNAs may function as sponge or ceRNAs of the down-regulated anti-proliferative miRNAs in hyper-proliferative smooth muscle cells.

**Table 3:** List of up-regulated lncRNAs/mRNAs with potential down-regulated miRNA targets in cocaine and Tat treated hyper-proliferative smooth muscle cells.

LncRNA	Fold change	P-value	Micro RNA	No. of binding sites on lncRNA	Fold change	P-value
TCONS_00001909	1.631	0.037	hsa-miR-376b-5p	22	−3.855	0.029
			hsa-miR-193a-3p	7	−3.381	0.002
			hsa-miR-370-3p	6	−2.299	0.001
TCONS_00028198	1.552	0.018	hsa-miR-376b-5p	15	−3.855	0.029
			hsa-miR-33a-3p	13	−2.98	0.012
			hsa-miR-185-5p	21	−2.865	0.007
ENST00000585387	1.735	0.011	hsa-miR-491-5p	2	−2.585	0.041
			hsa-miR-185-5p	2	−2.865	0.007
			hsa-miR-654-5p	1	−1.787	0.019
TCONS_00022282	1.573	0.017	hsa-miR-185-5p	3	−2.865	0.007
TCONS_00025369	2.04	0.007	hsa-miR-491-5p	1	−2.585	0.041
TCONS_00012383	1.73	0.013	hsa-miR-185-5p	1	−2.865	0.007
			hsa-miR-503-5p	1	−4.321	0.0035
ENST00000595383	1.78	0.007	hsa-miR-1277-5p	1	−1.709	0.0145
			hsa-miR-33a-3p	1	−2.98	0.012
TCONS_00024430	1.77	0.015	hsa-miR-324-3p	2	−2.066	0.0391
ENST00000412357	1.54	0.048	hsa-miR-33a-5p	2	−3.541	0.0001
ENST00000429843	1.83	0.015	hsa-miR-146a-5p	1	−3.066	0.0023
miRNA	mRNA					
hsa-miR-1277-5p	APCS, LINC01554, NPPC, PLN, PTGER3, SERTM1, SPRR2F					
hsa-miR-146a-5p	CCR9, IL36, IRF5, NDNF, S100A12, TLR9, TMEM100					
hsa-miR-185-5p	VAT1L,UBXN10,UBE2QL1,SSX7,SMARCC2,SLC36A1,SLAMF6,SET,S100A7A, RNASE2, PRR23B, PLN,PLCB2, PIANP, PCDHA10, PABPC1L2A, NKAIN1,LRRC38,LINC01554,KIR3DL3, KCNK9, KCNJ15, HK2, GPR12, FXYD4, ETV3L, DNAH10OS, DMBX1, DLX2, CXCL16, C5AR1, BAK1, ARL5C, ANKRD63, VAT1L,					
hsa-miR-193a-3p	TP53AIP1, RHAG, PLCB2, PARP15, NOS1, NDOR1, MAGEE1, MAGEA3/MAGEA6, KRT33B, IQCJ, HTR1A, GABRP, CYP2S1, CRYAA/CRYAA2, CNTN2, BAK1					
hsa-miR-324-3p	ZDHHC22, TMEM86A, STS, SCGN, RAPGEFL1, RAB44, PTPRU, PTGER3 PRSS55, PRAMEF22, PPP4R4, NKAIN1, IGF2, FKRP, FAM92B, EFNA3 DUSP13, DNAH10OS, DMBX1, CYP2A6, ARL5C, ALDH3B2					
hsa-miR-33a-3p	TMEM69, P2RY8, HLA-DQB1, CDKL4, ASB18					
hsa-miR-33a-5p	TTC4, TMEM86A, PLN, GPR88					
hsa-miR-370-3p	UBXN10, TSNARE1, TMEM86A, SLAMF6, SDK2, NKAIN1, DNAH10OS, CRYAA/CRYAA2, CHRND, C10orf25					
hsa-miR-376b-5p	ZNF772, ZNF74, TMEM69, SERTM1, SELL, SCN11A, RP5_1052I52, PADI3, KRTAP26-1, KIAA0408, CHRNA4, CCR1, C16orf89, C10orf25, BTG4					
hsa-miR-491-5p	UROC1, UBXN10, TSNARE1, TMEM86A, TMEM69, TLR9, SMYD1, SLC36A1, SLC25A42, SLC22A12, SELL, S100A7A, RAX, RASGRP4, RAB44, PTPRU, PLCB2, PIANP, PCDHGB2, P2RY8, P2RX2, NOS1, NKAIN1, NCALD, KIR3DL3, KCNIP2, IGFL3, IGF2, HLA-DQB1, GLP1R, FKRP, ELF5, EFNA3, CYP2S1, CNTN2, CHAD, CA1, C5AR1, C1orf229, C1orf115, C17orf107, BAK1					
hsa-miR-503-5p	TMEM100, TAT, SLC36A1, KLHL26, IGF2, FKRP, FCGR1A, CYP2S1, BAK1 ARL5C, ALDH3B2					
hsa-miR-654-5p	VNN3, UBXN10, UBE2QL1, TSNARE1, TRIM55, TMEM86A, SLC25A42, RORC RGR, PEBP4, PABPC1L2A, OBP2B, MUC12, MMEL1, KIR3DL3, KCNIP2 GPR88, GPR17, GCM1, FXYD4, ETV3L, DUSP13, DRD2, DNAH10OS, DMBX1 CXCL16, CLEC12B, C17orf50, C17orf107					

Of the analyzed up-regulated lncRNAs, TCONS_00001909 had 22, 7, and 6 predicted binding sites for the down-regulated miRNAs, miR-376b, -193a and -370, respectively. This connection can potentially explain the role of this lncRNA in the up-regulation of mRNAs such as ZNF74, SCN11A, C10orf25, MAGEA3/MAGEA6, UBXN10 and TSNARE1 that are regulated by these miRNAs (Table 3). The lncRNA, TCONS_00028198 contains 15, 13 and 21 binding sites for the down-regulated miRNAs, miR-376b, -33a, and -185 respectively, which in turn is known to regulate several of the up-regulated mRNAs implicated in proliferation such as, VAT1L, UBE2QL1, SSX7, SLAMF6, S100A7A, RNASE2, PLN, PCDHA10, PABPC1L2A, CXCL16, LRRC38, FXYD4, ETV3L, ZNF74, SCN11A and C10orf25 (Table 3). The lncRNA, ENST00000585387 has two predicted binding sites each for the miRNAs, miR-491 and -185 and thus may act as a ceRNA for these miRNAs which are known to regulate the up-regulated genes, NOS1, KCNIP2, IGF2, ELF5, EFNA3, CHAD, C17orf107 and BAK1. Overall, evidence from our bioinformatic analysis suggests that many lncRNAs that are significantly up-regulated have the potential to sequester many anti-proliferative miRNAs in HPASMCs exposed to combined treatment of cocaine and Tat and thereby regulate the expression of their downstream target genes.

Our *in silico* analysis using down-regulated mRNA/lncRNA with up-regulated miRNAs also identified an inter-regulatory relationship between them. We identified 21 down-regulated lncRNAs that may potentially target 7 up-regulated miRNAs in cells treated with combined C + T treatment when compared with untreated controls. Based on prediction and experimentally validated findings, these miRNAs had a potential to target 131 down-regulated mRNAs in C + T vs control comparison (Table 4).

**Table 4:** List of down-regulated lncRNAs/mRNAs with potential up-regulated miRNA targets in cocaine and Tat treated hyper-proliferative smooth muscle cells.

LncRNA	Fold change	P-value	miRNA	No. of binding sites on lncRNA	Fold change	P-value
ENST00000596769	−2.8621	0.0043	hsa-miR-133a-3p	7	1.6677	0.0919
			hsa-miR-130b-5p	4	1.7023	0.0386
ENST00000603037	−1.5199	0.0208	hsa-let-7a-5p	4	2.1128	0.0011
TCONS_00014969	−3.2602	0.0194	hsa-miR-125b-5p	4	1.5854	0.0032
ENST00000589511	−2.6857	0.0467	hsa-miR-125b-5p	4	1.5854	0.0032
TCONS_00012168	−1.6284	0.0000	hsa-miR-130b-5p	4	1.7023	0.0386
TCONS_00026830	−4.1484	0.0010	hsa-let-7a-5p	3	2.1128	0.0011
ENST00000412149	−1.5780	0.0134	hsa-miR-10b-5p	3	1.6354	0.0038
			hsa-miR-125b-5p	3	1.5854	0.0032
ENST00000603095	−1.7070	0.0282	hsa-miR-133a-3p	3	1.6677	0.0919
ENST00000596769	−2.8621	0.0043	hsa-miR-10b-5p	2	1.6354	0.0038
TCONS_00026830	−4.1484	0.0010	hsa-miR-130b-5p	2	1.7023	0.0386
ENST00000595853	−2.6963	0.0011	hsa-miR-130b-5p	2	1.7023	0.0386
ENST00000569618	−2.0383	0.0213	hsa-miR-98-3p	2	1.7129	0.0391
ENST00000521148	−1.5589	0.0243	hsa-miR-10b-5p	1	1.6354	0.0038
ENST00000549516	−2.2653	0.0242	hsa-miR-125b-5p	1	1.5854	0.0032
ENST00000518355	−1.7250	0.0039	hsa-miR-125b-5p	1	1.5854	0.0032
ENST00000570084	−1.8786	0.0116	hsa-miR-130b-5p	1	1.7023	0.0386
ENST00000422345	−2.2413	0.0100	hsa-miR-133a-3p	1	1.6677	0.0919
TCONS_00013442	−1.8486	0.0223	hsa-miR-133a-3p	1	1.6677	0.0919
			hsa-miR-454-3p	1	2.2159	0.0003
miRNA	mRNA					
hsa-let-7a-5p	AC144568.2, ARHGEF15, ARHGEF39, ATXN1L, CACNG4, CBL, CCL3L1 CXorf36, DRD3, GJB4, HOXC11, IL12RB2, KCTD21, LIMD2, LOR, LRRC10 MS4A7, MYO5B, PADI4, PRLR, PRR18, SALL4, SCN4B, SCYL3, SLC13A3 SPRED3, TBC1D13, TMPPE, TNFSF9, TRIM67, TTC22, ZBTB8B, ZNF835 ZPLD1					
hsa-miR-125b-5p	ALOX5, ARHGEF39, CCDC169, CCR5, CD69, CTB_54O99, GLB1L2, ITGA7 KCNJ9, KCNK10, KCTD21, LIMD2, MALL, OVOL1, PLD6, PRAMEF18/PRAMEF19 RASD2, RASGRF2, SERPINE3, SHTN1, SLC25A48, ST8SIA4, TTPA, VTCN1 ZBTB8B					
hsa-miR-130b-5p	CCDC170, CCSER1, CEP83, ENDOU, KCNJ13, LHX6, OR2H1, RAET1L, RNF138 SLC26A4, SMOC1, SYN3, ULBP2					
hsa-miR-133a-3p	ARHGEF39, BMP7, CACNA2D4, CELF6, COL9A2, DAND5, DOLPP1, GLOD5 GZMM, KCNJ9, PIRT, PROK1, PRR30, RASGRF2, SH3GL2, SIGLEC8, SLC7A2 TDRKH, ZNF69					
hsa-miR-454-3p	CD69, CDK19, ELK3, FAM129C, GRK7, KCNK10, MCHR1, MS4A7, NMUR2 ODF4, PLPPR1, RASGRF2, RD3, RGS7BP, SMOC1, ST8SIA5, ZPLD1					
hsa-miR-10b-5p	ANXA13, AWAT1, CHST9, DAND5, FCMR, IL5RA, LHPP, NPAS3, PLA1A, PRAMEF18/PRAMEF19, SLC6A19, SLC7A2, SYNGR1, ZNF69					
hsa-miR-98-3p	AC144568.2, ALG10B, C20orf197, P2RY13, RAD51B, RNF138, SLC25A31 ST8SIA4, ZC2HC1B					

Next, we validated the expression of the lncRNAs, TCONS_00001909, TCONS_00028198 and ENST00000585387 along with four of their corresponding predicted miRNA targets: miR-376, -193, -185 and -491 (Fig. 6A), using qRT-PCR. These three lncRNAs were up-regulated in qRT-PCR by 2, 1.8 and 3.6 -fold, respectively, which was in concordance with the microarray data. The expression of these selected CeRNAs in C + T treated cells was significantly up regulated not only when compared to untreated cells, but also when compared to mono-treated cells with cocaine or Tat. All four target miRNAs validated by qRT-PCR were down regulated in cocaine + Tat treated cells compared to other treatments (Fig. 6B).

### Involvement of lncRNAs ENST00000585387 and ENST00000495536 in cocaine and Tat mediated smooth muscle hyperplasia

**Figure 7:**
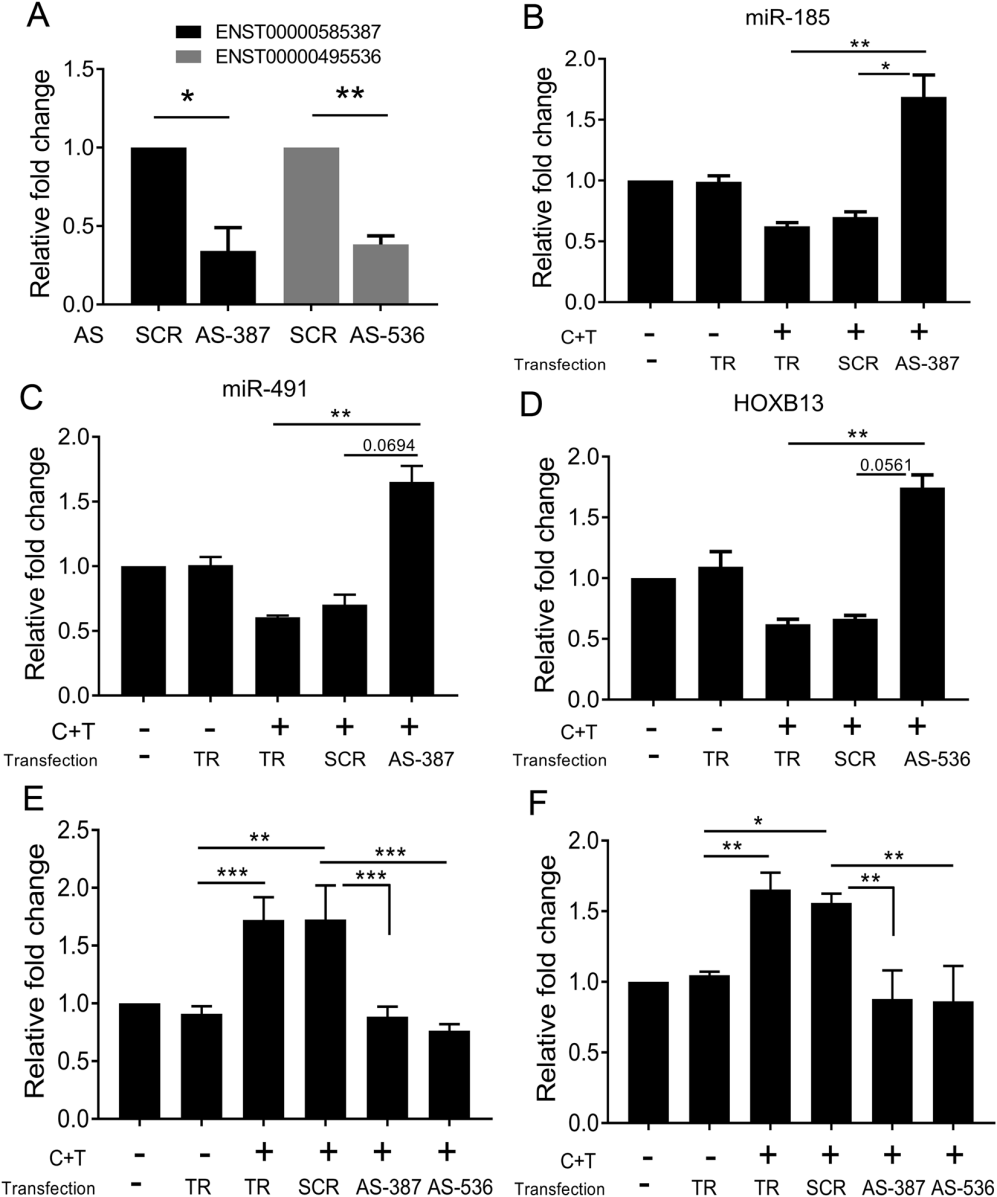
Knock down of selected up-regulated lncRNAs alleviates the cocaine and Tat induced smooth muscle proliferation. (**A**) Validation of knock down of lncRNA. (**B**,**C**) Levels of miR-185 (B) and -491 (C) in HPASMC with and without knockdown of ENST00000585387. (**D**) Levels of HOXB13 mRNA in HPASMC on knockdown with ENST00000495536. (**E**) MTS and (**F**) CyQuant proliferation assays on HPASMCs transfected with antisense oligonucleotides against target lncRNA. AS-antisense oligonucleotide gapmeR, SCR-scrambled gapmeR, AS-387 against ENST00000585387, AS-536 against ENST00000495536, TR-transfection reagent control and C + T-combined treatment of cocaine and Tat. *p*-value ^***^<0.001, ^**^<0.01, ^*^<0.05.

Next, we selected the lncRNA ENST00000495536 that was associated with the gene, HOXB13 and the lncRNA ENST00000585387 premised to act as a ceRNA for miRNAs, miR-491, -185, for examining of their role in smooth muscle proliferation. We utilized gapmeR based LNAs to knockdown the expression of these two lncRNAs in HPASMCs. As shown in Fig. 7A, a 50% reduction in the levels of both lncRNA was observed, with a 40 nM concentration of antisense oligonucleotides (ASO) compared to HPASMCs transfected with scrambled gapmeRs (Fig. 7A). Both MTS and Cyquant proliferation assays revealed that the knockdown of either, ENST00000495536 or ENST00000585387, results in the inhibition of cocaine and Tat induced proliferation of HPASMCs compared to un-transfected or, scrambled gapmeR transfected C + T treated cells (Fig. 7E,F). These changes in proliferation were also clearly visible under the microscope as shown by the representative phase contrast images in Supplementary Fig. S1. Concomitant to these findings we observed that the knockdown of lncRNA 387 or 536 also prevented the C + T mediated down-regulation of miR-185 and -491 or HOXB13 mRNA (Fig. 7B,C). Together, these results suggest the role of lncRNAs in the cocaine and Tat mediated hyper proliferation of HPASMCs.

## Discussion

Dys-regulated expression of miRNAs, mRNAs and lncRNAs has been reported to play a critical role in the progression of PAH^38,39^. However, an integrated analysis of the potential interactions between these different types of RNA molecules is only beginning to emerge^34,40^. In this study, we predicted the inter-regulatory relationship between lncRNAs, mRNAs and miRNAs and identified their role in hyper-proliferation of pulmonary smooth muscle cells. We utilized high throughput techniques such as microarray and small RNAseq to quantify the expression levels of mRNAs, lncRNAs and miRNAs. Our analysis of dys-regulated mRNAs and lncRNAs, as described above, revealed a number of lncRNA - mRNAs associations involved in regulating hyperplasia of HPASMCs on exposure to cocaine and Tat protein. The KEGG pathway analysis of the significantly dys-regulated mRNAs identified neuroactive ligand receptor signaling and Ras signaling among the top signaling pathways. The combined analysis of dys-regulated miRNAs, mRNAs and lncRNAs revealed the potential of lncRNAs to act as ceRNAs/sponges for many anti-proliferative miRNAs leading to up-regulation of pro-proliferative mRNAs in cocaine and Tat treated HPASMC. Overall, these analyses provided clues to the possible cis and trans acting roles of various up-regulated lncRNAs in hyper-proliferative smooth muscle cells by inter-regulating, either directly or indirectly, transcriptional or post-transcriptional expression of downstream proliferative genes.

Recent research on lncRNAs is proving to be a key to unlocking many underlying molecular mechanisms of development and pathology of diseases^41^. A vast number of lncRNAs have been identified that still remain to be characterized. Adding to the complexity of understanding lncRNAs is their versatile nature of cellular regulation, making them both intriguing and challenging to be characterized^42^. Lately, many studies have identified multiple lncRNAs implicated in PAH^39,43–46^, however, an integrated study considering miRNAs, mRNAs and lncRNAs with relevance to HPASMC hyper-proliferation and PAH have been lacking.

The pathway analysis we performed on dys-regulated mRNAs identified many factors playing active role in neuroactive ligand receptors and ras signaling. It is interesting to note that genes such as C5AR1, CHRNA4 and HTR1A from the neuroactive ligand receptor pathway have been reported in association with PAH, lung cancer, COPD and idiopathic pulmonary fibrosis^47–49^. On the other hand, Ras signaling molecules such as FGFR2, PLA2G2A and PLA2G5 have been shown to be involved in vascular remodeling, idiopathic pulmonary fibrosis (IPF) and chronic thromboembolic pulmonary hypertension (CTEPH)^50–52^. Analyzing lncRNAs associated with molecules in these highly enriched/activated pathways in cocaine and Tat induced HPASMC can help us understand their mechanistic roles in these diseases.

One of the top up-regulated vascular disease related lncRNAs in HPASMCS treated with both cocaine and Tat was ENST00000495536. It is classified as an intergenic lncRNA located around 12,000 bp, in the antisense strand, from the HOXB13 gene, PRAC2 small nuclear protein and miRNA 3185. The gene, PRAC2, was not expressed in our mRNA microarray data, however the expression of HOXB13 was significantly down-regulated in cocaine and Tat treated cells in the microarray data, and was confirmed by qRT-PCR. Loss of HOXB13 in colorectal tumor cells has been associated with the increase in proliferation^53,54^. The knock down of HOXB13 associated lncRNA, ENST00000495536 in HPASMC prevented the cocaine and Tat mediated decrease in HOXB13 mRNA expression with the corresponding inhibition of smooth muscle hyper-proliferation. The possibility of an association between the lncRNA, ENST00000495536 and the gene, HOXB13 is further strengthened by the observation that in most cases, the lincRNAs function in trans- while antisense lncRNAs function in cis, to modulate gene expression^55^. However, the mechanistic association of HOXB13 and its nearby lncRNA, ENST00000495536 in HPASMC and PAH needs to be further established.

Our analysis identified cancer as one of the top diseases associated with the significantly differentially expressed lncRNAs and mRNAs and revealed the integrated link between the gene/lncRNA pairs: CBL/T216482 and GDF7 (BMP12)/T185733, prominently associated in cancer pathways. The expression of both the lncRNA, T216482 and its associated mRNA, CBL were down-regulated in cocaine and Tat treated HPASMCs. The CBL gene has been reported to be decreased in primary bone tumors, and ectopic CBL expression has been reported to reduce bone tumorigenesis by promoting tyrosine kinase receptor degradation^56^. Enhanced CBL activity is known to result in the down-regulation of EGFR expression and inhibition of proliferation in colon tumor cells^57^. GDF7 also known as BMP12 is reported to be involved in tissue regeneration^58,59^. It’s in proximity to not only lncRNA T185733 but also to two other lncRNAs, NR_046836 and ENST00000602736. Furthermore, RND1 located up-stream of the up-regulated intergenic lncRNA, TCONS_00020413 is known to act as a tumor suppressor by attenuating Ras/MAPK signaling^60^. Its expression was reduced in response to the combined treatment of cocaine and Tat.

Analysis of significantly dys-regulated miRNAs and their mRNA targets in cocaine and Tat treated cells revealed many up-regulated miRNAs including miR-133 and miR-125 that target mRNAs involved in proliferative signaling. Of these two miRNAs, miR-133 is known to play role in vascular stress, remodeling and cell survival^61^. whereas miR-125 has been reported to play a role in pulmonary hypertension with increased expression observed in the lungs of hypoxic animals^62^. LncRNAs are known to regulate gene expression by sponging miRNAs that regulate mRNAs^63^. Our combined bioinformatics analysis of the up-regulated lncRNA and mRNAs, along with the down-regulated miRNAs, in cocaine and Tat cells revealed a number of lncRNA that possessed binding site for these down-regulated anti-proliferative miRNAs. We found that the lncRNA, TCONS_00028198 contained biding sites for miRNAs, miR-376b, -33a, and -185, and the lncRNA, ENST00000585387 contained bind sites for miRNA, miR-185 and -491, making them potential regulators of these miRNAs. Of these miRNAs, miR-376b and miR-33a have been shown to be significantly down-regulated in PH^64,65^ and miR-185 has been shown to mediate lung epithelial cell death post oxidative stress^66^. In addition, multiple studies suggest miR-185 and miR-491 as suppressors of cell- proliferation including vascular smooth muscle cells^67–70^. We observed that the knock down of lncRNA 397 results in up-regulation of miR-185 and miR-491 in HPASMCs exposed to the combined treatment of cocaine and Tat and this correlated with the decrease in C + T mediated hyper-proliferation of cells. Another significantly up-regulated lncRNA, TCONS_00001909 contained potential binding sites for miRNA, miR-193a, implicated in PAH^71^ and miR-370, recently identified as a tumor suppressor^72^.

Many of the up-regulated mRNA targets of these miRNAs have not yet been characterized in either smooth muscle proliferation or in PAH however some of the mRNAs namely, CXCL16 and LRRC38 are known to play a positive role in proliferation^73,74^. The up-regulated gene, IGF2, which is a predicted target of miR-185, has been reported to be up-regulated in cells having a hypoxic environment^75^. Furthermore, it is intriguing to note that some of the alterations in the expression of lncRNAs, mRNAs and miRNAs may be attributed to a regulatory mechanism(s) operative at the DNA level considering that we observed the association between the down-regulated histone deacetylase-4 (HDAC4) mRNA and up-regulated miR-10^76^ in C + T treated cells. It’s plausible that miRNA dependent changes in the expression of HDAC proteins, known to play a critical role in the chromatin condensation and transcriptional repression^77,78^ may in turn be involved in regulating the expression of lncRNA,miRNA and mRNA levels.

Long noncoding RNAs such as NEAT1, NRON, lincRNA-p21 and PANDA are known to modulate HIV infection and replication^79–81^. However to the best of our knowledge, this is the first attempt to report the effect of viral proteins in the interplay between lncRNAs, mRNAs and miRNAs. Although the functions of lncRNAs characterized here are based on their primary sequence, there are reports that suggest that the tertiary structure of lncRNAs play a major role in deciding lncRNA function^82^. Therefore, further functional evaluation of the identified lncRNA, miRNA and mRNA associations is needed for a comprehensive mechanistic understanding of their role in smooth muscle hyper-proliferation and pulmonary vascular remodeling.

## Materials and Methods

### Cell culture and RNA isolation

Primary human pulmonary arterial smooth muscle cells (HPASMC) were grown on smooth muscle cell media (SMCM) with growth factors, 2% fetal bovine serum and penicillin/streptomycin until 70% confluency on 6 well plate and made quiescent for 48 h with serum free media and then treated with either cocaine (C) at 1 µM final concentration or with Tat (T) protein at 25 ng/mL concentration or with both (C + T) for 12, 24 and 48 h based on our previous findings^12,21^. Cells were harvested in TriZol reagent and the total RNA was isolated as per the manufacturer recommended protocol. Considering that HIV-PAH is more prevalent in males particularly among intravenous drug users^1,83^ pulmonary arterial smooth muscle cells used for this study were from males.

### Microarray analysis for lncRNA and mRNA expression

The total RNA isolated from HPASMC were quantified by the NanoDrop ND-1000 and RNA integrity was assessed by standard denaturing agarose gel electrophoresis. For microarray analysis, Agilent Array platform was employed. The sample preparation and microarray hybridization were performed based on the manufacturer’s standard protocols with minor modifications. Each sample was amplified and transcribed into fluorescent cRNA along the entire length of the transcripts without 3’ bias utilizing a random priming method (Array Star Flash RNA Labeling Kit, Arraystar Inc). The labeled cRNAs were hybridized onto the Human LncRNA Array v4.0 (8 × 60 K, Arraystar). After washing the slides, the arrays were scanned by the Agilent Scanner G2505C.

Agilent Feature Extraction software (version 11.0.1.1) was used to analyze the acquired array images. Quantile normalization and subsequent data processing were performed using GeneSpring GX v12.1 software package (Agilent Technologies). The statistical significance of the differentially expressed LncRNAs and mRNAs between cocaine and Tat (C + T) group and control group were calculated using the Student’s two sample t-test. The cutoff for significance was set at an absolute fold-change ≥1.5 and p-value ≤0.05. Biological functions of the significantly differentially expressed genes were obtained from the KEGG (Kyoto Encyclopedia of Genes and Genomes; http://www.genome.jp/kegg) and GO (Gene Ontology; http://www.geneontology.org) databases/online-software. The data were also analyzed through the use of IPA (QIAGEN Inc., https://www.qiagenbioinformatics.com/products/ingenuitypathway-analysis) for the analysis of associated pathways. The statistical significance of the association of genes with a biological function or pathway was calculated using the right-tailed Fisher’s Exact Test.

### Small RNA sequencing

The global unbiased miRNA profiles of HPASMCs treated with cocaine and/or Tat were interrogated using illumina’s small RNA sequencing technology (*TruSeq Small RNA Sample Prep Kit*). Samples were prepared in triplicates (n = 3, for each treatment group). The small RNA-seq samples were sequenced for 50-cycle single end reads using a HiSeq2500 Sequencing System (*Illumina*, *San Diego*, *CA*). The 12 samples were multiplexed in a single lane. The sequence yield of the lane was approximately 375 million reads providing enough sequencing depth for each small RNA-Seq sample.

The small RNA-Seq reads with non-canonical letters (e.g. N) were first removed from the samples. Adaptors were clipped from the remaining reads. Resulting reads that were shorter than 17 bp were discarded. The remaining reads were mapped to the human genome (GRCh38.rel77) using Bowtie^84^. The read abundance estimates of all known human miRNAs (mirBase v21) were computed using miRDeep2^85^.

The miRNA expression counts obtained from this process were analyzed using a negative binomial generalized linear model (NB-GLM) from the edgeR package^86^ to identify statistically significant differentially expressed miRNAs between the control and the three treatment conditions, cocaine (C), Tat (T) and combined treatment of cocaine and Tat (C + T). The edgeR package implements advance empirical Bayes methods to estimate feature-specific biological variation under minimal levels of biological replication. Finally, Hierarchical Clustering (Euclidean distance and Ward’s linkage) was performed to show the distinguishing clusters of miRNA expression patterns among different sample groups.

### Integrative analysis of dys-regulated non coding RNAs and mRNAs

Differentially expressed mRNAs, miRNAs and lncRNAs that were more than 1.5 fold up or down regulated between treatments groups (C + T vs. Con) with a p-value less than 0.05 were considered for integrated analysis. The association between significantly differentially expressed miRNAs and mRNAs were explored using the IPA software. The analysis was performed, first by only considering experimentally validated miRNA/mRNA interactions without including microarray mRNA data (Fig. 5B); and then by including microarray mRNA data to also consider experimentally validated and highly predictive miRNA/mRNA interactions (Fig. 5C). The IPA software uses information on experimentally validated miRNA/mRNA interactions from TarBase and miRecords. It uses information on predicted miRNA/mRNA interactions from TargetScan. IPA also maintains a repository of miRNA related information from peer-reviewed literature that it uses for its analysis. The regulatory relationships between differentially expressed miRNAs and lncRNAs were obtained from LncBase v2.0 using the DIANA Tools software^37^. Only experimentally validated interactions were considered. Both IPA software and DAVID (Database for Annotation, Visualization and Integrated Discovery) software^87^ were used to perform pathway enrichment analysis.

Significantly differentially expressed lncRNAs were also annotated for biological functions and diseases using several databases. In particular, the LncRNADisease database^88^ provided a source of experimentally validated and predicted lncRNA-disease associations, including a separate list of predicted lncRNA association with cardiovascular function and disease, and a list of lincRNA disease associations predicted by co-expression analysis. This database also provided a curated list of experimentally supported lncRNA interactions with mRNA. We used data from the lncRNA-Cancer gene co-expression network (lnCaNet)^89^ to identify genes that are putatively co-expressed with lncRNAs based on expression correlation. Information from the Lnc2Cancer database^90^ was used to obtain experimentally validated associations between lncRNA and human cancer.

### Quantitative Real Time (qRT)-PCR

The cDNA was prepared using RT^2^ First strand synthesis kit (Cat#330404, Qiagen) as per the manufacturer’s protocol. qRT-PCR was performed using RT^2^ SYBR Green ROX qPCR Master Mix kit (Cat#330522, Qiagen). The qRT-PCR primers for the selected mRNA and lncRNAs were either custom designed using PrimerQuest tool and obtained from Integrated DNA technologies or ordered form Qiagen. Tables S1 and S2 (Supplementary File II) lists details of all the primers used in this study.

### GapmeR antisense oligonucleotide transfections

Antisense oligonucleotide (ASO) based gapmeRs were designed against lncRNA using Qiagen, gapmer designer tool. The top ranked ASO were ordered along with positive and negative controls. HPASMC were reverse transfected while seeding in 6 well plate at 2 × 10^5^ cells/well with specific gapmeR ASO at 40 nM using HiPerfect transfection reagent (Cat#301704; Qiagen). After 24 h of transfection, the cells were serum starved  for 24 h and  then treated for 48 h with cocaine and Tat. RNA isolation and qRT-PCR were performed as described above to evaluate the knockdown of specific lncRNAs. For proliferation assay cells were reverse transfected in a 96 well plate with a cell density of 10^4^ cells/well. Proliferation assay was performed as described earlier^9^. Proliferation of the cells was quantified using CellTiter 96® AQueous One Solution Cell Proliferation Assay (MTS) (Promega,G3582) and CyQUANT® Cell Proliferation Assay Kit (Invitrogen, C7026) as recommended by the manufacturers.

### Statistical analysis

The statistical analyses of the qRT-PCR data as shown in Figs 4C,D, 6A,B and 7A–D were carried out using the normalized data obtained relative to control. We used Kruskal Wallis test to assess if differences exist among the treatment groups followed by Dunn’s test for pairwise comparisons. The multiple testing adjustments were carried out using Benjamini and Hochberg’s method. Since our interest was also to assess the treatment effects of cocaine and Tat separately as well as combined cocaine and Tat (as a single group) with respect to untreated control we later used one factor analyses model (Kruskal Wallis test) where the Factor is the treatment effects. In this way, we were able to assess the individual treatment effects as well as the synergistic effect of the combined treatments. We did not design the study as a two factor model because we were not assessing the interaction effect of the treatments. The statistical analyses for the proliferation assay data as shown in Fig. 7E,F was carried out similarly on the background corrected raw readings. After the completion of the statistical analyses, for the better visual clarity of the outcomes, the data in the figures were presented in the relative scale as compared to control by calculating the fold changes. Since the control group was the reference for the fold change calculation, its value was set to 1 in the bar plots and hence lack the error bars. The test results were considered significant if the multiple tests adjusted P values were ≤ 0.05.

## Supplementary information

Supplementary File II

Supplementary File I

## Data Availability

The datasets generated during and/or analyzed during the current study are available from the corresponding author on reasonable request.
