# Supplementary material for: Analysis of lncRNA-miRNA-mRNA Interactions in Hyper-proliferative Human Pulmonary Arterial Smooth Muscle Cells
Source: Sci Rep. 2019 Jul 19;9:10533. doi: 10.1038/s41598-019-46981-4 (PMC6642142; doi:10.1038/s41598-019-46981-4)
Supplement: Supplementary file 1 — Supplementary File II [file 41598_2019_46981_MOESM1_ESM.docx]

**Supplemental Material**

**Analysis of lncRNA-miRNA-mRNA Interactions in Hyper-proliferative Human Pulmonary Arterial Smooth Muscle Cells.**

Mahendran Chinnappan^1^, Sumedha Gunewardena^2,3^, Prabhakar Chalise^4^, Navneet K. Dhillon*^,1,2^

^1^Division of Pulmonary and Critical Care Medicine, ^2^Department of Molecular & Integrative Physiology, ^3^Kansas Intellectual and Developmental Disabilities Research Center, ^4^ Department of Biostatistics, University of Kansas Medical Center, Kansas City, Kansas , USA.

*Correspondence and requests for reprints should be addressed to Navneet K. Dhillon, Division of Pulmonary and Critical Care Medicine, Department of Medicine, Mail Stop 3007, University of Kansas Medical Center, 3901 Rainbow Blvd, Kansas City, KS 66160, Tel: (913) 945-6018, Fax: (913) 588-4098, Email: [ndhillon@kumc.edu](mailto:ndhillon@kumc.edu)

**Table S1.** Primers custom designed/ordered from IDT* for qRT-PCR validation of lncRNAs, mRNAs

| **Serial No.** | **Primer Name** | **Forward (5’to 3’)** | **Reverse (5’to 3’)** |
| --- | --- | --- | --- |
|  | ENST00000495536 | GCCTTTGCTTCCTTCCATTTC | AGCCAGAGAGTGAGCAATAAC |
|  | NR_108042 | TGTGTAGTACGACAGATCAATGG | TTCTGAGCCTCGCTGAAAC |
|  | TCONS_20413 | GGACCAAACTTTCCTAGCTGAA | CCTTTGGTGGTGTGCTACTT |
|  | NR_046836 | CATGCTCTCCTGGGCTTAC | CTGCATCACCGTAGTGACTC |
|  | ENST00000602736 | GAACATCTGGAATGCGCTTG | GTTTCTCCACCCAGAGATCAT |
|  | T185733 | ACTTCAGACTCCTGGTACAGA | CCAACATGCCTGGAAGCT A |
|  | NR_027022 | CAGTCAGACCAGCAGTGAATAG | GGGATTCTCTTGATGGACAGTG |
|  | HOXB13 | GTTACCAGTCTTGGGCTCTC | GCAAATGCTGCCTTCCAAA |
|  | BDKRB1 | TTTGGGAGGACTTCATTGACC | CCGGCCCACAAAGACATAA |
|  | CBL | GTCATTGCCCAGAACAACATC | CCAACTCACTGGTCCTCTAAAC |
|  | RND1 | GCGACTCGGATGCAGTATTA | GGGTGCTGGGACAATAATCTA |
|  | GDF7 | CACCAACCATGCCATCATTC | TGTCCTCGTATTGCTTGTAGAC |
|  | GOLT1A | CATCATTGGCCTGAGGAAGA | CAGCGTAGGAGCACGATAAC |
|  | TCONS_00001909 | GCCTATACAGCAGAACTACCCT | AGGCAGTGTAGACATGGTGG |
|  | TCONS_00028198 | GGACCCTCCCCACAGTTAAA | AGCTGCAGACCTTTTTGAGGA |
|  | ENST00000585387 | CCACCAGACAGAGCAGGATG | TCTTCCACAAGGGATGGAATG |

*Integrated DNA Technologies

**Table S2.** Primers obtained from Qiagen for qRT-PCR validation of miRNAs and mRNAs

| **Serial No.** | **Primer Name** | **Cat. No** | **Company** |
| --- | --- | --- | --- |
|  | HPRT1 | PPH01018C-200 | Qiagen |
|  | miR-376 | MS00045164 | Qiagen |
|  | miR-193 | MS00007581 | Qiagen |
|  | miR-185 | MS00003647 | Qiagen |
|  | miR-491 | MS00004326 | Qiagen |
|  | U6 | MS00014000 | Qiagen |


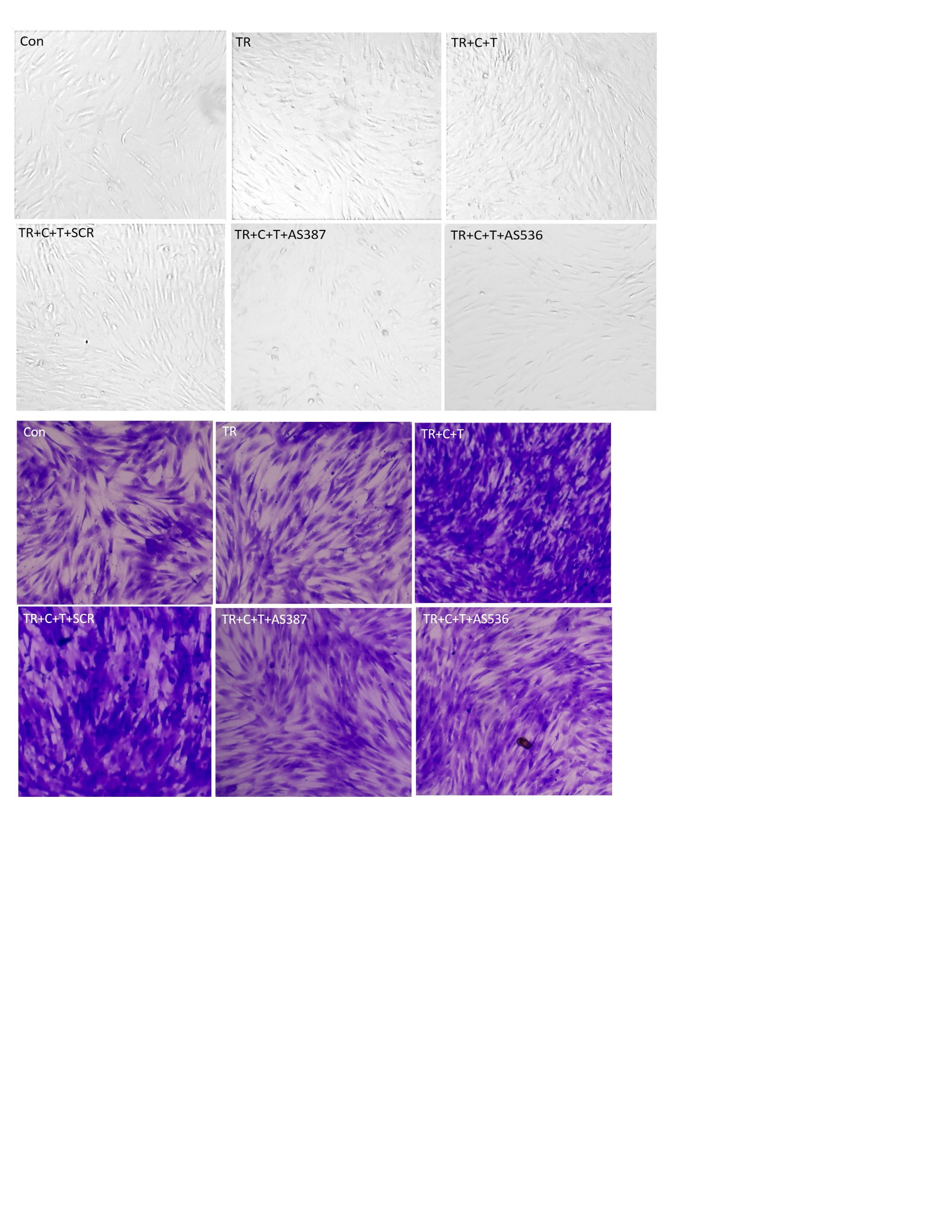


**Figure S1**. Phase contrast microscope images (20x) showing decreased proliferation of HPASMC with with specific knock down of the lncRNAs ENST00000585387 (AS387) and ENST00000495536 (AS536) using reverse transfection of antisense oligonucleotide gapmeRs. AS-antisense oligonucleotide gapmeR, SCR-scrambled gapmeR, TR-transfection reagent control and C+T-combined treatment of cocaine and Tat.
